# RNA binding proteins co-localize with small tau inclusions in tauopathy

**DOI:** 10.1186/s40478-018-0574-5

**Published:** 2018-08-01

**Authors:** Brandon F. Maziuk, Daniel J. Apicco, Anna Lourdes Cruz, Lulu Jiang, Peter E. A. Ash, Edroaldo Lummertz da Rocha, Cheng Zhang, Wai Haung Yu, John Leszyk, Jose F. Abisambra, Hu Li, Benjamin Wolozin

**Affiliations:** 1 Department of Pharmacology and Experimental Therapeutics, Boston University School of Medicine, Boston, MA USA; 2 Mayo Clinic, Rochester, MN USA; 3 Department of Pathology and Cell Biology, Taub Institute for Alzheimer’s Disease Research, Columbia University Medical Center, New York, NY USA; 4 University of Massachusetts Medical Center, Worcester, MA USA; 5 Sanders-Brown Center on Aging, Department of Physiology, Spinal Cord and Brain Injury Research Center, and Epilepsy Center, University of Kentucky, Lexington, KY USA; 6 Department of Neurology, Boston University School of Medicine, Boston, MA USA; 7 Department of Pharmacology and Neurology Program in Neuroscience, Boston University School of Medicine, 72 East Concord St., R614, Boston, MA 02118-2526 USA

**Keywords:** Alzheimer’s disease, Neurofibrillary tangle, Immunohistochemistry, Mass spectrometry, Protein interactome, Protein aggregation

## Abstract

The development of insoluble, intracellular neurofibrillary tangles composed of the microtubule-associated protein tau is a defining feature of tauopathies, including Alzheimer’s disease (AD). Accumulating evidence suggests that tau pathology co-localizes with RNA binding proteins (RBPs) that are known markers for stress granules (SGs). Here we used proteomics to determine how the network of tau binding proteins changes with disease in the rTg4510 mouse, and then followed up with immunohistochemistry to identify RNA binding proteins that co-localize with tau pathology. The tau interactome networks revealed striking disease-related changes in interactions between tau and a multiple RBPs, and biochemical fractionation studies demonstrated that many of these proteins including hnRNPA0, EWSR1, PABP and RPL7 form insoluble aggregates as tau pathology develops. Immunohistochemical analysis of mouse and human brain tissues suggest a model of evolving pathological interaction, in which RBPs co-localize with pathological phospho-tau but occur adjacent to larger pathological tau inclusions. We suggest a model in which tau initially interacts with RBPs in small complexes, but evolves into isolated aggregated inclusions as tau pathology matures.

**Electronic supplementary material:**

The online version of this article (10.1186/s40478-018-0574-5) contains supplementary material, which is available to authorized users.

## Introduction

RNA binding proteins (RBPs) have emerged as major factors in a number of neurodegenerative disorders, including tauopathies [31, 32]. RBPs represent a large class of proteins responsible for RNA metabolism, regulating key events including mRNA maturation, trafficking, and eventual translation. Importantly, many RBPs have been shown to have strong genetic links to neurodegenerative disease; for example, mutations in TDP-43, FUS, hnRNPA1, and ATXN2 have all been shown to cause amyotrophic lateral sclerosis (ALS), frontotemporal lobar degeneration (FTLD) and/or spinocerebellar ataxia [24]. Other RBPs have also been implicated in AD, Huntington’s disease, and Creutzfeldt-Jakob disease [24].

Following these initial discoveries, evidence now suggests that transient aggregation mediated by low-complexity domains within RBPs is crucial for the involvement of these proteins in disease. RBPs typically contain a “prion-like”, low complexity glycine-rich domain in their sequences, which regulate their self-aggregation [21, 35]. This regulated aggregation is important for RBP function, as many RBPs regulate the formation of a variety of ribonucleoprotein (RNP) granules. Such granules, which include stress granules (SGs) and P-bodies, are responsible for the transient recruitment and aggregation of mRNA transcripts for varying purposes [29, 32]. SGs recruit mRNA into granules for storage and protection following translational stress, while P-bodies recruit mRNA for eventual degradation [29, 32]. Many disease-linked RBPs are proteins involved in the formation and maintenance of SGs.

In particular, recent findings have shown that T-cell intracellular antigen 1 (TIA1) and other RBPs interact with hyper-phosphorylated tau and accumulate in tandem with tau pathology in diseases such as Alzheimer’s disease (AD) and frontotemporal dementia (FTD) [37, 38]. RBPs also regulate splicing of MAPT exon 10, which provides an additional mechanism for control of tau aggregation by determining whether the tau transcript codes for 3 or 4 microtubule repeats [9, 18, 40]. The aggregation of tau is a major pathological hallmark of these diseases, where the microtubule associated protein tau becomes hyperphosphorylated, and mislocalizes in the soma [41]. Once in the soma, tau begins to aggregate, eventually forming large neurofibrillary tangles and resulting in neurodegeneration. Recently, our lab demonstrated that tau interacts with TIA1 in SGs, which increases in sarkosyl insoluble aggregates. The association of tau with TIA1 and SGs also was shown to promote tau-mediated degeneration of primary hippocampal neurons [37]. This phenotype can be rescued by TIA1 knockdown in primary neurons [37]. We proceeded to show that TIA1 reduction (haploinsufficiency) is also protective in vivo, reducing neurodegeneration, rescuing cognition and increasing survival in the PS19 mouse model of tauopathy [1]. These results point to an essential role for TIA1 and SG biology in the pathophysiology of tauopathy.

We now present an enhanced view of the interaction of RBPs in the pathophysiology of tauopathy. We have used a proteomic approach to identify multiple RBPs that have significantly altered associations with tau as pathology develops in the rTg4510 mouse model of tauopathy including multiple splicing factors. The rTg4510 mouse model of tauopathy inducibly expresses P301L 4R0N tau under control of a tetracycline promoter, and when aged in the absence of doxycycline develop pathology by 4–5 months [30]. We also present optimized immunohistochemical methods for detecting RBPs in pathological tissues. We combine the optimized immunohistochemical methods with biochemical methods to validate the proteomic findings. We identify specific RBPs that associate with pathological phospho-tau early in disease and become increasingly insoluble as disease progresses. Together our results provide important evidence indicating that RNA binding protein pathologies are a major feature of tauopathy.

## Results

### TIA1 colocalizes with phosphorylated tau in tauopathy

Recent work from our laboratory demonstrates that TIA1 and other SG markers colocalize with pathological tau in Tg4510 and PS19 mouse brains as well as human post-mortem AD and FTDP-17 brain samples [1, 37, 38]. Immunohistochemical identification of TIA1 inclusions in pathological samples has proven to be particularly difficult, with some observing TIA1 inclusions, and others unable to observe inclusions [8, 11, 15]. While this could represent differences in the types of cases examined, we have also observed TIA1 reactivity to be highly sensitive to methodological conditions.

To improve reproducibility among laboratories, we set out to define optimal conditions for TIA1 immunohistochemistry. We began by determining optimal conditions for fixation of mouse tissue. rTg4510 tissues were drop-fixed in 4% buffered paraformaldehyde for 24 or 48 h, then transferred to 30% sucrose, incubated at 4 °C for 48 h, sectioned and subjected to immunohistochemistry. Shorter fixation times produced much stronger reactivity for both TIA1 and NeuN, which is also a RNA binding protein (Additional file 1: Figure S4). The 24 h fixation protocol was used for all subsequent experiments using animal tissues. We also optimized conditions to reduce background. Our previous studies used Sudan black to quench background fluorescence, however such quenching also has the drawback of reducing antibody signals [23, 38]. Photobleaching proved to provide much more effective quenching of autofluorescence without significant loss of antibody reactivity (Additional file 1: Figure S5). Application of shorter fixation times and photobleaching significantly improved the sensitivity for detecting RBPs in tissue samples.

**Fig. 1:**
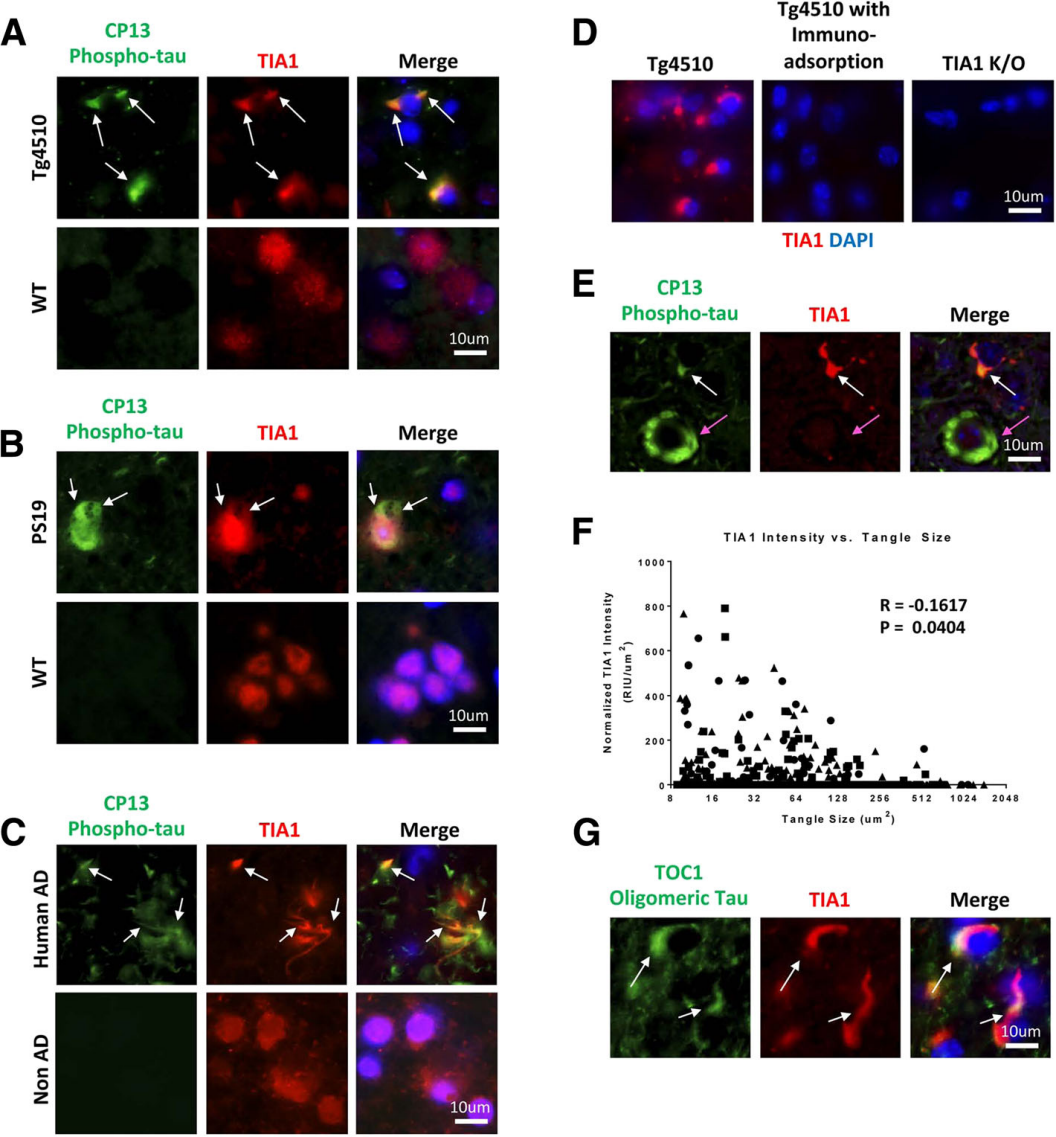
TIA1 preferentially colocalizes with phosphorylated tau that is not in NFTs. Immunohistochemical analysis of the stress granule nucleating protein TIA1 (red) shows colocalization with phosphorylated tau (CP13 antibody against pSer202, green) in rTg4510 (**a**), PS19 (**b**) and human AD tissues (**c**) with DAPI in blue. **d** Immunohistochemistry of TIA1 on rTg4510 with and without immunosorbtion using TIA1 peptide, as well as TIA1 staining of TIA1 knockout mouse tissue, also show that the TIA1 antibody used for this analysis is specific for TIA1 and does not have off-target staining. **e** Further analysis of TIA1-tau colocalization in rTg4510 tissues indicates that TIA1 primarily has cytoplasmic colocalization with smaller tau aggregates (white arrow) over larger NFT-like tau aggregates (pink arrow). This is quantified in (**f**) using Imaris Bitplane software, where cytoplasmic TIA1 intensity negatively correlates with the size of tau tangles in neurons (*R* = − 0.1617 with a two tailed *p* value of 0.0404). **g** TIA1 also colocalizes with oligomeric tau stained using the TOC1 antibody specific for oligomeric tau

Next we compared commercially available antibodies for immunohistochemical reactivity. We observed that the anti-TIA1 antibody from Abcam (Abcam cat#40693) gave the strongest results (Fig. 1, Additional file 1: Figure S1). Using the Abcam antibody, we consistently observed TIA1 colocalization with CP13 positive phospho-tau in the rTg4510 and the PS19 mouse models of tauopathy (Fig. 1a-c). The strength of reactivity varied with lot number (Fig. 1e), and thus the work in this manuscript used lot GR151575 (Fig. 1e). Reactivity with antibodies from other vendors did not work as well as the Abcam antibodies (Additional file 1: Figure S1). In all cases, specificity of the TIA1 reactivity was demonstrated by the absence of anti-TIA1 reactivity observed following immuno-adsorption or staining of TIA1 knockout brain tissue (Fig. 1d). These results demonstrate that immunohistochemical reactivity with the Abcam anti-TIA1 antibody is bona-fide TIA1 reactivity.

We proceeded to characterize how the co-localization of TIA1 and tau varied with the type of tau pathology. Analysis of patterns of co-localization in 6 month-old rTg4510 brain tissue demonstrated a distinct correlation of TIA1 co-localization with the size of CP13-positive tau inclusions (Fig. 1e, f). Abundant co-localization was observed with small CP13 reactive puncta, while little co-localization was observed with large fibrillar CP13 positive tau inclusions (Fig. 1e). Previous results from our laboratory indicate that TIA1 selectively interacts with oligomeric tau [1]. To test whether the small TIA1 reactive inclusions contained tau oligomers, we probed the tissues with the anti-tau oligomer antibody TOC1 [19]. Strong co-localization was observed between anti-TIA1 and the TOC1 tau oligomer specific antibodies (Fig. 1f, R = − 0.1617, *p* = 0.0404). From this we conclude that TIA1 preferentially associates with small tau inclusions containing oligomeric tau.

### Other RNA binding proteins change in their association to tau in tauopathy

**Fig. 2:**
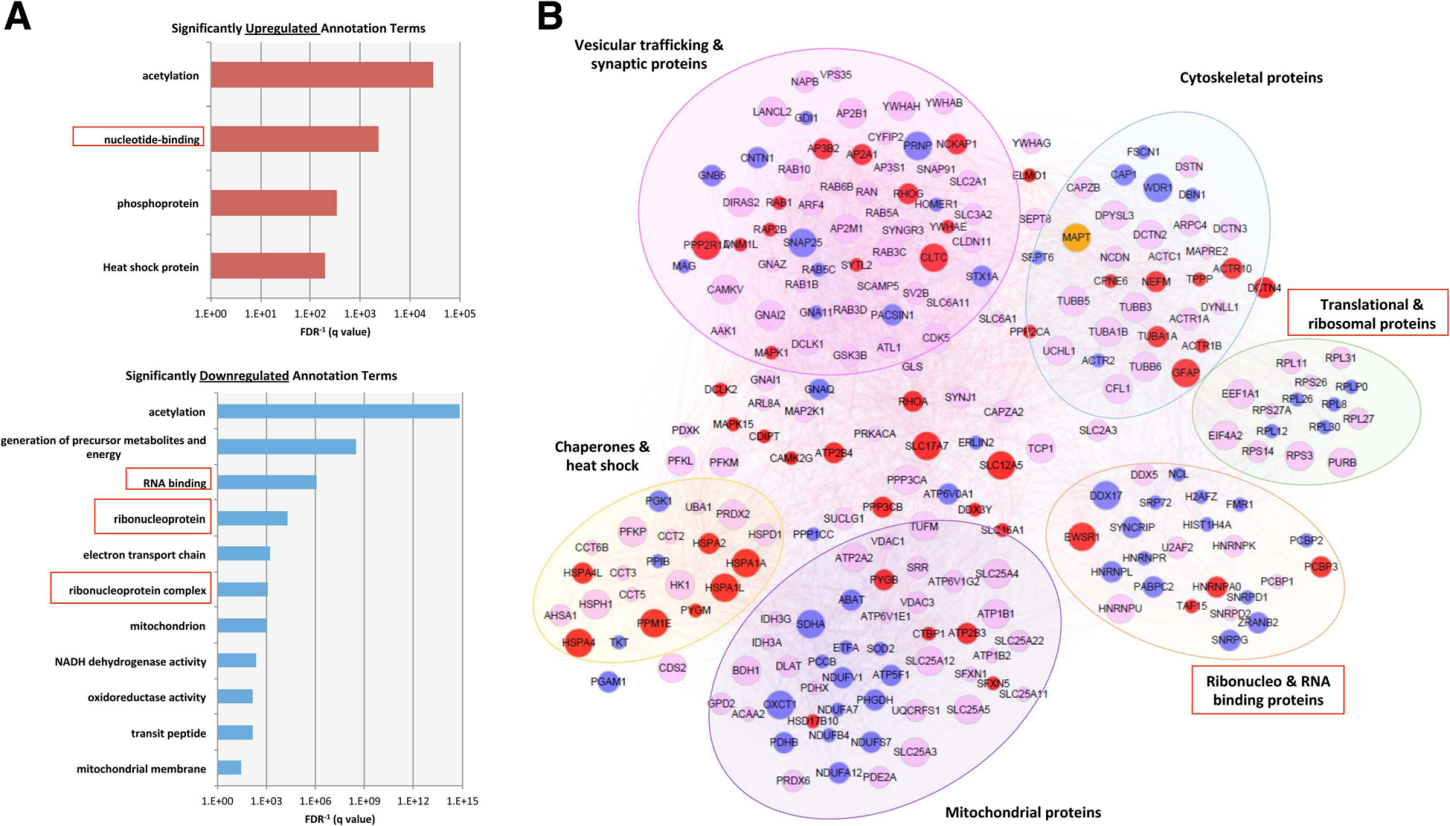
RNA binding proteins change in their association to tau in tauopathy. Using Tau13 antibody, tau was immunoprecipitated from the cortex of either 2.5 month old induced or uninduced rTg4510 brains.The co-immunoprecipitating proteins were subsequently identified by mass spectrometry. A variety of nucleotide binding and ribonucleoproteins show differential association to tau in rTg4510 tissue compared to WT tissue as indicated by significantly upregulated and downregulated annotation terms (**a**). Part (**b**) represents a network diagram of proteins enriched in the tau interactome. Red nodes denote proteins whose association with tau is ≥5-fold increased in Tg4510 vs uninduced control. Blue nodes denote proteins whose association with tau is ≥5-fold decreased in Tg4510 vs control. Node size is proportional to degree of replication (*N* = 4), non-replicating proteins excluded. Key proteins include EWSR1, HNRNPA0, PABP, PCBP2, DDX5, TAF15, FMR1, RPL and RPS family members, and eIF4a2

The association of phosphorylated tau with the stress granule protein TIA1 raises the possibility that other RBPs and ribosomal proteins might also interact with tau [1, 25, 26, 37]. To explore the tau interactome, we immunoprecipitated (IP) total human tau from 2.5 month old Tg4510 mouse frontal cortex expressing low or high amounts of human tau protein due to inclusion or exclusion of doxycycline from the mouse chow, respectively; IPs were performed using the Tau13 antibody, which is a high affinity antibody that selectively recognizes total human tau (independent of tau phosphorylation state) [5]. We also did IPs from MAPT −/− mice to provide negative controls for the IPs. This design allowed exploration of neurons exposed to relatively low or high amounts of stress, but preceding a phase with extensive neurodegeneration. The tau complexes were then analyzed using orbitrap mass spectrometry to identify proteins in the tau interactome (Fig. 2); any proteins pulled down by the Tau13 antibody from the MAPT−/− mice were removed from tau interactome observed in the rTg4510 IPs. This analysis identified a large number of proteins associated with tau, including RBPs, translational and ribosomal proteins, cytoskeletal proteins, chaperones, heat shock proteins and synaptic proteins (Fig. 2 and Additional file 2: Table S1).

Comparing the tau networks from the induced and un-induced rTg4510 mice revealed striking changes. Multiple proteins showed decreased association with tau in the 2.5 m induced rTg4510 mice. Proteins linked to RNA metabolism comprised 3 of the top 6 GO annotations categories of decreased proteins (Fig. 2a, bottom panel). Interaction of tau with some of these proteins, including HNRNPA0, HNRNPK, eIF4a, and RPL11, was validated by immunoprecipitating tau from 6 month old PS19 P301S tau mouse brains; these mice overexpress mutant P301S tau at 5 times endogenous levels (Additional file 1: Figure S2). Confirmation is also provided by comparison to our prior study showing the requirement of tau for the presence of many proteins in the TIA1 interactome network [37]. These changes corresponded to large-scale changes in the interaction of tau with these particular groups of proteins. For instance, 65% of proteins in the RBP group showed decreased association with disease; in contrast, only 16% of proteins in the synaptic and vesicular protein group showed decreased association with disease (Fig. 2a).

The groups of proteins whose interaction with tau increased with disease were equally striking. Heat shock proteins/chaperones and phospho-proteins were 2 of the 3 groups of proteins upregulated in the 2.5 m induced rTg4510 mouse cortex (Fig. 2a, top panel), with 37% of heat shock or chaperone proteins showing increased association with tau with disease (Fig. 2a). This upregulation is consistent with the stress associated with pre-tangle stage that predominates in the 2.5 m rTg4510 mouse [30]. Interestingly, the third group of proteins whose association with tau increased in the 2.5 m mice was nucleotide binding proteins; these include RBPs such as EWSR1, TAF15 and HNRNPA0, which we previously reported to co-localize with tau pathology in the rTg4510 model of tauopathy [37] (Fig. 2a, b). In addition, aggregation-enhancing mutations in EWSR1 and TAF15 are associated with ALS, suggesting that changes in the biology of these proteins are sufficient to drive neurodegenerative disease [6, 7]. Taken together, these results highlight that complexes containing tau and RBPs are a major feature of the pathophysiology of tauopathy.

### RNA binding proteins become insoluble in tauopathy

**Fig. 3:**
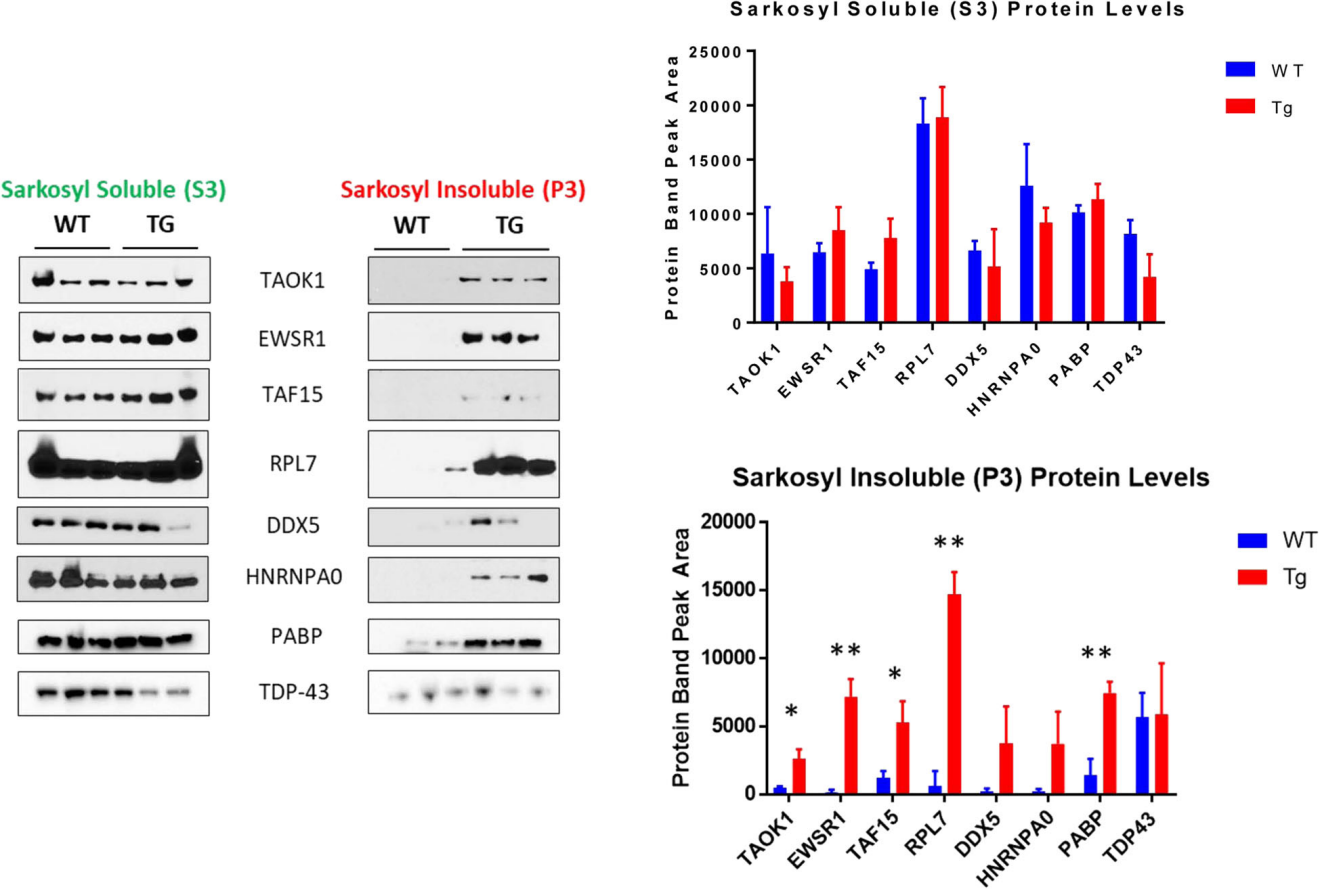
RNA binding proteins become insoluble in the cortex of rTg4510 mice. (**a**, **b**) Immunoblots of the sarkosyl soluble (S3) and insoluble (P3) fractions isolated from rTg4510 cortical tissues indicate that many RBPs become insoluble as tau pathology develops. The fractions were also probed for TDP-43, which is not associated with tau aggregation. Quantification of these immunoblots (**c**, **d**) shows statistically significant RBP accumulation in the P3 fraction of induced rTg4510 mouse cortex using a two-tailed t-test (*p* = 0.00599 for TAOK1; *p* = 0.0007599 for EWSR1; *p* = 0.0122 for TAF15; *p* = 0.000252 for RPL7; *p* = 0.00195 for PABP; *p* = 0.0926 for DDX5; *p* = 0.0638 for HNRNPA0)

A key feature of RBP biology is the formation of transient, dynamic complexes, such as stress granules, P-bodies, transport granules, and even splicing complexes. With time or in disease, many RBPs exhibit a tendency to develop into insoluble aggregates. To explore whether RBPs become insoluble in mouse models of tauopathy, we fractionated frontal cortex from 8 m old rTg4510 mice aged with low or high levels of human tau (i.e., ± dox). The samples were then biochemically separated into sarkosyl soluble or insoluble fractions and immunoblotted. Levels of RBPs in the sarkosyl soluble group did not change with disease state (Fig. 3a, b). In contrast, the amount of RBP present in the insoluble fraction was elevated in diseased tissues for most of the RBPs examined (Fig. 3a, b). Analysis of TDP-43 levels showed no change with disease state, consistent with the lack of association of this protein with tau aggregation processes. (Fig. 3a, b). These results suggest that a range of RBPs form insoluble aggregates with disease in tauopathy.

### RNA binding proteins colocalize with phospho-tau but not mature neurofibrillary tangles in tauopathy

**Fig. 4:**
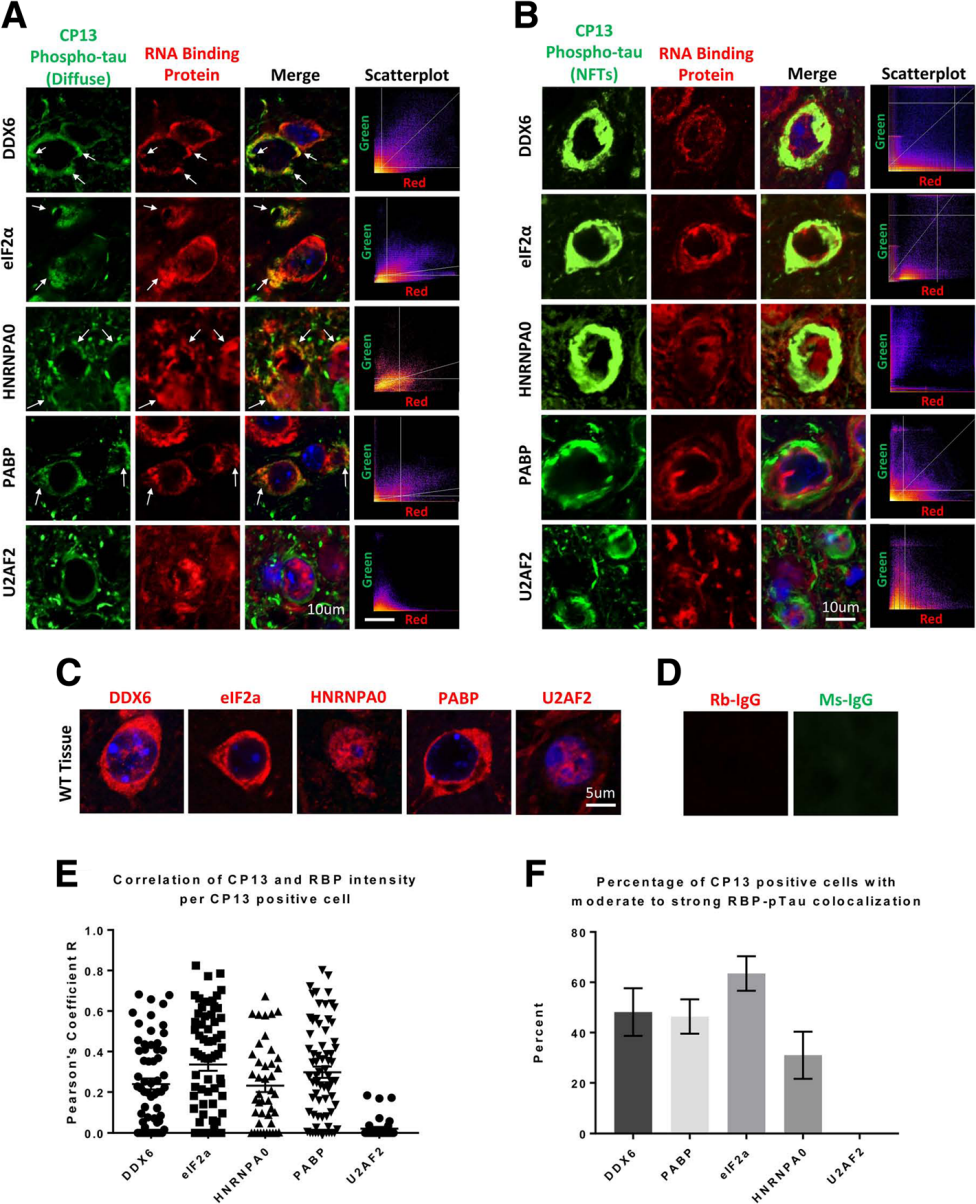
RNA binding proteins show significant colocalization with diffuse phospho-tau but not NFTs in the rTg4510 cortex. (**a**) Immunohistochemical analysis of rTg4510 tissue (*n* = 3) has also revealed a significant colocalization in the cortex between the RBPs DDX6, PABP, HNRNPA0, and eIF2a (red) with pathological phospho-tau stained using the CP13 antibody (green). However, the RBP and splicing factor U2AF2 does not show significant correlation. To the right of each merged image is a scatterplot of the pixel intensities for each pixel of the image in the red channel vs. the green (Pearson correlation coefficients *r* = 0.773 for DDX6, 0.791 for eIF2α, 0.325 for HNRNPA0, 0.798 for PABP, and − 0.14 for U2AF2). This colocalization is greatly reduced and/or completely lost as tau aggregates into large NFTs which are brightly fluorescent and fill the cell bodies of neurons (**b**) (*r* = 0.069 for DDX6, 0.372 for eIF2α, 0.481 for PABP, − 0.03 for HNRNPA0, and − 0.009 for U2AF2). **c** Staining of wild-type C57Bl/6 mice also indicates that HNRNPA0 is predominantly nuclear in healthy animals, while the rTg4510 staining shows significant cytoplasmic localization of HNRNPA0 (**a**, **b**). (**d**) Negative controls IHC using rabbit and mouse normal IgG indicates that there is no off target staining or fluorescence in our tissues. **e** Pearson coefficients of correlation between CP13 positive tau with RBPs DDX6, eIF2α, HNRNPA0, PABP, and U2AF2 are graphed for individual neurons using ImageJ. For all cases except U2AF2, neurons show heterogeneity in colocalization between phospho-tau and the RBPs stained, from no colocalization to fully overlapping reactivity patterns in individual neurons. The percent of neurons with *r* > 0.3 is graphed in (**f**) as the percentage of neurons showing moderate to strong correlations between green:red intensity (DDX6 = 36% of neurons; eIF2α = 54% of neurons; HNRNPA0 = 35% of neurons; PABP = 33% of neurons; U2AF2 = 0% of neurons)

Using immunohistochemistry with confocal microscopy, we proceeded to examine how deposition of RBPs in disease correlated with tau pathology. We examined proteins identified by mass spectrometry in the tau network (Fig. 2), proteins that we had previously shown to associate with tau, as well as proteins thought to be involved in the RNA translational stress response and stress granule formation. Frontal cortex from 6 month old rTg4510 mice was used for the study because of the presence of both mature and evolving tau pathology at this age. Imaging of RBPs using fluorescence immunohistochemistry showed the accumulation of RBPs co-localized with or near diffuse deposits of phosphorylated tau identified with CP13, an antibody recognizing pS202 (Fig. 4, Additional file 1: Figure S3). The RBPs and proteins linked to RNA metabolism primarily colocalized with phosphorylated tau present in neuronal somas (Fig. 4a); scatterplots done on the images demonstrated that when overlap was present there was strong co-localization with tau pathology (Fig. 4a, e). We quantified the fraction of neurons exhibiting CP13 reactivity that also exhibited RBP reactivity (Fig. 4f, g). Robust correlation for CP13/RBP co-localization was observed for DDX6, eIF2α, hnRNPA0 and PABP, but not for U2AF2 (Fig. 4f, g); robust correlation was also observed for TIA1 (Fig. 1f). Interestingly, little colocalization was observed with mature NFTs showing bright condensed CP13 reactivity (Fig. 4b), consistent with the studies of TIA1 in Fig. 1e, f. Some of the RBPs (e.g., DDX6 and hnRNPA0) appear to remain as aggregates but accumulate adjacent to the tau NFTs, as if the RBPs are excluded from large, consolidated tau tangles, and pushed to the periphery of such tangles (Fig. 4b).

**Fig. 5:**
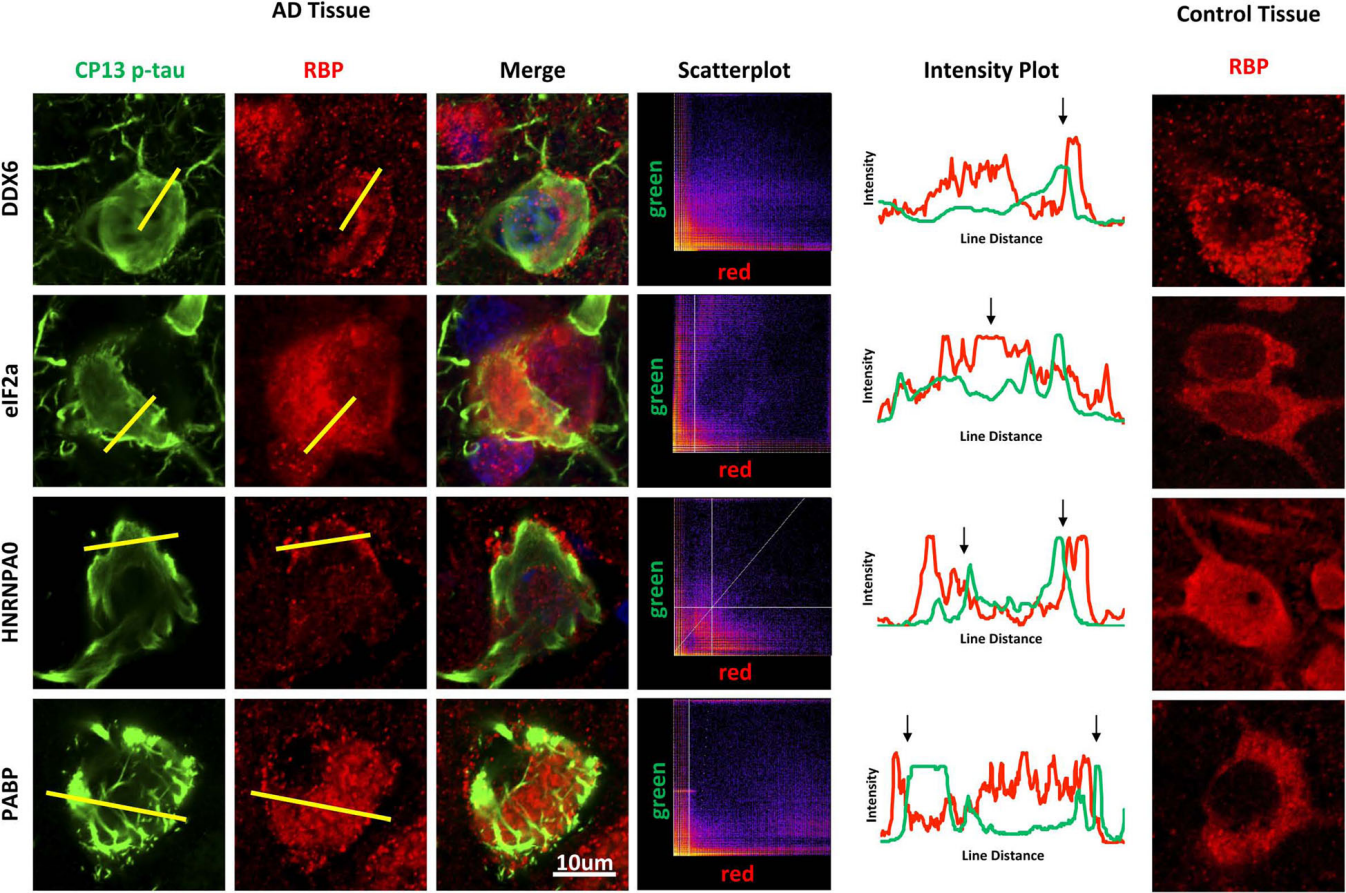
RBPs show granularization and interfacing with NFTs in late stage human AD tissue. Immunohistochemical analysis of human AD frontal cortex tissue (*n* = 6) shows that RBPs (red) and NFTs stained with CP13 tau (green) do not colocalize (*r* = 0.001 for DDX6; 0.176 for eIF2α; 0.031 for HNRNPA0; and 0.222 for PABP). However, trace analyses across lines within the image (yellow bars) indicate that peak fluorescent intensities (reported as fluorescence intensity plots over the distance of the line for tau in green and the RBP in red) between phospho-tau and multiple RBPs are immediately adjacent to each other, indicating protein interfacing or interaction between the edges of NFTs and RBPs

Finally, we examined the pattern of reactivity of RBPs in human tissue. We examined temporal cortex of late Braak stage V and VI human AD patients (Fig. 5). At this stage of disease, CP13 positive phospho-tau exists predominantly as NFTs (showing bright condensed CP13 reactivity), with little diffuse phospho-tau, such as is seen at 6–8 months of age in the rTg4510 mouse model (Fig. S6). RBP inclusions were readily apparent in the human tissues using antibodies against DDX6 and hnRNPA0 (Fig. 5). Comparison of the distribution of the RBP and tau deposits in individual neurons suggests an inverse correlation between RBP localization and mature NFTs similar to that observed in the rTg4510 mouse tissue (Fig. 5, intensity plots). In addition, we observed that some RBPs (e.g., DDX6 and hnRNP0) accumulated as pathological inclusions, but accumulated adjacent to the mature tangles, much like what was observed in rTg4510 mice (Fig. 5). These results demonstrate that deposits of RBPs occur near deposits of pathological tau, but suggest that RBPs are excluded from the aggregated tau as the deposits consolidate.

## Discussion

Characterization of the tau interactome in the rTg4510 mouse model of tauopathy reveals striking disease-related changes in interactions between tau and multiple RBPs. About 65% of proteins in the RBP group show decreased tau association with disease. A smaller number of RBPs increase the association of tau with disease, including EWSR1, TAF15 and hnRNPA0, each of which have been linked to ALS (e.g., EWSR1 or TAF15) or have related family members linked to ALS (hnRNPA0 shares homology with hnRNPA2B1). Formation of aggregates was evident biochemically and by immuhistochemisty which is consistent with prior studies showing multiple U1 spliceosome components that accumulate in the sarkosyl-insoluble fraction of AD tissues [2].

Imaging studies suggest that the pattern of co-localization varied depending on the type of RBP, and the size of the inclusion. For the RBPs examined in this manuscript, TIA1 showed the strongest co-localization with tau pathology. Co-localization for TIA1 with tau could be observed for phospho-tau (CP12, p-S202) and oligomeric tau (TOC1). Other RBPs, including hnRNPA0, DDX6, eIF2α and (to a lesser extent) PABP also co-localized with phospho-tau pathology. Direct co-localization was observed with small tau puncta, but for large tau inclusions these RBPs tended to accumulate immediately adjacent to the inclusion. The observation of RBP inclusions adjacent to tau tangles has also been observed for small nuclear ribonuclear proteins, such as U2AF2 [12, 13]. The differential localization of RBP inclusion with tau aggregate size suggests a model in which these proteins initially co-localize with tau but become excluded as the tau aggregates consolidate into mature neurofibrillary tangles. This work adds key information to our prior data indicating that phospho-tau (specifically, tau phosphorylated at the classic proline directed serine/threonine sites associated with pathology) promotes SG formation [37].

**Fig. 6:**
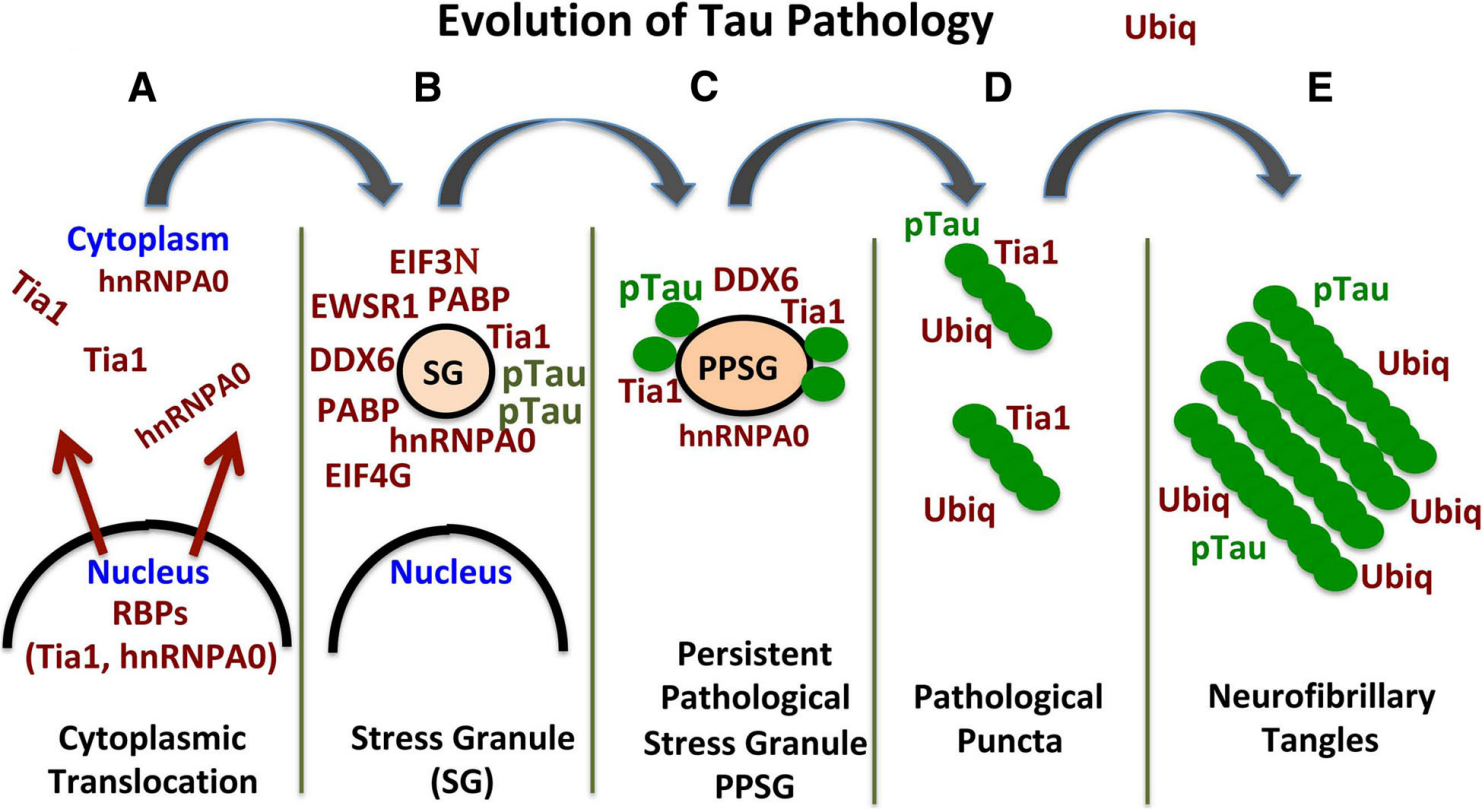
Model for the evolution of tau pathology **a** Stress elicits translocation of nuclear RBPs, such as TIA1 and HNRNPA0, to the cytoplasm where they distribute diffusely in the cytoplasm. **b** Core nucleating RBPs, such as TIA1, coalesce to form SGs, in a process that is stimulated by tau oligomers. Secondary nucleation brings in other RBPs, such as EWSR1, PABP, DDX6, EIFs, etc. **c** Persistent stress (such as occurs in disease) causes particularly insoluble RBPs (and tau) to consolidate to form persistent pathological stress granules. More soluble RBPs separate from the persistent pathological SG, and disperse without aggregating. **d** The tau oligomers evolve into fibrils forming pathological puncta, which contain classic markers of pathology such as hyper-phosphorylation and ubiquitination [43]. Other RBPs that remain associated with the persistent pathological SG, begin to form aggregated puncta around the tau puncta. **e** Tau fibrillizes forming neurofibrillary tangles, which are relatively inert, do not act in the translational stress response and contain few RBSs

These results suggest the hypothesis that tau and RBPs exhibit progressive maturation (Fig. 6). This process appears to begin with a stress response that includes the translational stress response, which leads to cytoplasmic translocation of nuclear RBPs (Fig. 6a), followed by formation of functional SGs in part through interaction with phosphorylated, oligomeric tau (Fig. 6b) [1, 37]. The association of tau with SGs also produces aggregation, much as occurs with disease-prone RBPs during extended or repetitive periods of liquid liquid phase separation (LLPS) [22, 27, 28, 37]. Tau also undergoes LLPS, and does so in a manner that is accelerated by the presence of mRNA [14, 39, 42]. The similar biophysical behavior of tau and RBPs, the association of tau with SGs, the ability of tau to promote SG formation, and the tendency of tau to aggregate in the presence of SGs, provide a strong basis for a model based on the interaction of tau with SGs. Co-localization of tau with SG-associated RBPs suggest that the tau complexes with RBPs complexes that either are SGs or resemble SGs, which then mature during chronic stress into persistent pathological SGs (Fig. 6c). Based on our data, we hypothesize that persistent SGs lose many types of RBPs and perhaps reducing their physiological activity (Fig. 6d); ubiquitin is added into the model at this point to reflect the well-documented association of ubiquitin with mature tangle pathology [43]. As the aggregated tau in these pathological SGs consolidates they appear to exclude many RBPs, and ultimately become the inert pathological structures that are referred to as neurofibrillary tangles (Fig. 6e). Meanwhile, the excluded RBPs form inclusions that accumulate adjacent to the neurofibrillary tangles. The latter stages of this hypothesis (steps 6c - e) remain to be explicitly tested beyond the correlative evidence presented above, but the model sets up a paradigm to guide future studies.

This model is further supported by accumulating evidence pointing to a persistent translational stress response as a key pathway leading to the accumulation of SGs. Chronic disease could produce a chronic stress, which leads to persistence of SGs. The high concentration of RBPs in SGs (100–400-fold higher than dispersed RBP levels) creates conditions that also promote aggregation of RBPs into insoluble amyloids, which over time accumulate [16, 24, 31]. Support for the persistent SG hypothesis comes from protection experiments in cell culture and in transgenic models. Bonini and colleagues demonstrated that chemical inhibition of the SG pathway rescues the ALS phenotype in drosophila [20]. Ataxin-2 deletion, which also inhibits the SG/translational stress response pathway, also delayed disease progression in a mouse model of ALS [3]. The relevance of the SG pathway to tauopathy was recently demonstrated by our observation that TIA1 reduction protects against disease progression in a mouse model of tauopathy [1]. Studies using primary neurons support these results by demonstrating that both RNAi knockdown of TIA1 and chemical inhibition of the SG pathway are able to prevent tau-mediated toxicity [37]. Thus, multiple independent lines of evidence demonstrate that RBPs, SGs and the translational stress response contribute to the pathophysiology of tauopathy and other neurodegenerative diseases.

The putative role for tau in regulating the RNA metabolism is supported by proteomic studies of tau interactomes from multiple different groups, which also identify similar classes of proteins that associate with tau. Immunoprecipitation and mass spectrometry of tau (both WT and P301L) binding proteins from SH-SY5Y neuroblastoma cells showed strong overlap of RBPs and ribosomal protein with the proteins identified in our study, including EWSR1, DDX5 & 17, hnRNPK, L, R and U, as well as ribosomal proteins RPL7, 8, 27 and 30 [10]. Proteomic studies of complexes containing tau from the rTg4510 mouse model and human AD tissues also report RNA binding and nucleotide binding proteins in their results, as well as multiple heat shock proteins and chaperones [4, 25]; work from the Abrisambra laboratory also directly demonstrates that tau over-expression inhibits RNA translation [25, 26]. Studies using HeLa cells report multiple RBPs in the tau interactome, including TIA1, hnRNP family members, and many ribosomal subunit proteins, and also demonstrated the presence of aggregated RBPs in the AD brain [36]. A recent study of tau-associated proteins in lymphoblastoid cell lines containing AKAP9 mutations linked to AD show enrichment of RNA binding and spliceosomal proteins in the tau proteome [17]. These proteins again include EWSR1, TAF15, DDX family members, and RPL family members, which parallels our findings. Finally, our own work previously demonstrated the association of TIA1 with tau by both proteomic analysis as well as immunoprecipitation [37]. These data demonstrate that identification of complexes containing tau and RBPs is a reproducible observation. The nature of the tau species responsible for binding to each RBP remains to be determined, and might differ between tau monomers, oligomers and fibrils; for instance, our recent study suggest that TIA1 exhibits preference for tau oligomers [1]. The studies also provide strong support for an emerging consensus that tau functions in stress to regulate the translation stress response through interaction with RBPs and SGs.

Our current study combines with accumulating prior studies to suggest that tauopathies (including AD) exist in the spectrum of neurodegenerative diseases and myopathies that are associated with dysfunction of RBPs, which already includes ALS and FTD. The current work advances the field by identifying a number of RBPs that can be reliably associated by immunohistochemistry with tau pathology, and providing optimized methods for detecting the association. These advances will facilitate rigor and reproducibility for this emerging field. Finally, the consistent role of RBPs and SGs in the mechanisms of multiple neurodegenerative diseases suggests that dysfunction of RBPs, SGs and translational stress response pathways plays a fundamental role in the pathophysiology of neurodegenerative diseases, and that seemingly disparate diseases might converge on common downstream mechanisms for neurodegeneration.

## Materials and Methods

### Animal husbandry and tissue collection

Animal husbandry for the rTg4510, PS19 and TIA1−/− mice was approved and performed as previously described in each indicated reference. For all brain harvesting, mice were anesthetized in an isoflurane chamber and perfused with ice cold PBS. Brains were then harvested and processed according to each subsequent experiment recorded below.

### Human brain samples

Temporal cortex tissue from human brain was used for the immunohistochemical studies. The samples were de-identified. The cases are listed in Table 1 below:

**Table 1:** AD cases used for immunohistochemical studies

Age	Gender	Braak Stage
74	M	V
96	M	V
57	F	VI
80	F	VI
97	F	V
87	F	VI
87	F	II
67	F	II
84	F	I
70	M	I
90	F	II
93	F	II
91	M	II
79	M	VI
95	M	II

### Immunohistochemistry and quantification

All mouse (*n* = 8 rTg4510 and n = 8 wild type C57BL6/J) and human (*n* = 7 AD cases and *n* = 8 aged control) tissue was sectioned at 20um on a cryostat and stained as free floating sections using Netwell baskets (VWR Cat#29442–132) in 12 well Falcon plates (Cat#353043). Extracted mouse tissues were drop fixed in15mL 4% PFA for 24 h, transferred to 15 mL 30% sucrose in PBS for 48 h, then sectioned and stored in cryoprotectant (30% ethylene glycol; 30% glycerol; 40% PBS) at − 20 °C. We note that a recent study of stress granule pathology showed excellent labeling of TIA1 and other RBPs in a mouse model of tauopathy using perfusion with cold paraformaldehyde (4%), followed by drop fixation in cold paraformaldehyde for 2 h, transfer to 30% sucrose in PBS for at least 48 h [33]. Human tissues were fixed and stored in periodate-lysine-paraformaldehyde (PLP) until sectioned using a Leica VT1200S vibratome. To quench lipofuscin autofluorescence, sections were photobleached under a 1500 lm white LED bulb for a minimum of 72 h at 4 °C while suspended in PBS with no lid or covering [34]. Note that the photobleaching is time-limited; sections examined were observed to recover lipofuscin autofluorescence after approximately 1 week following the photobleaching. Sections were then washed 3× in PBS for 30 s each wash followed by a 5-min incubation in detergent media (TBS with 0.25% Triton-X). Human tissue, but not mouse tissue, was then incubated in 1%*w*/*v* sodium borohydride (NaBH_4_; Sigma-Aldrich Cat#452882-25G) in PBS for 45 min to quench aldehyde autofluorescence which results from the over-fixation of tissue. All tissue was then incubated for 1 h in citrate based antigen unmasking solution (Vector Cat#H-3300) at 95 °C, with the exception of human TIA1 staining (Fig. 1), which was done using 0.05% citraconic anhydride (Sigma-Aldrich Cat#125318-25G) for 1 h at 95 °C. Tissue was blocked with gentle rotating for 2 h in detergent media with 5% donkey serum (Sigma-Aldrich D9663-10 mL) in PBS. After blocking, tissue was washed once in PBS for 30 s to remove excess detergent, then moved into 24 well plates with 200uL of the appropriate primary antibody dilutions and no basket overnight at 4 °C. To prevent evaporation, the lids of each plate were lined with a damp Kimwipe. Antibody catalog numbers and dilutions are recorded in Additional file 3: Table S2.

Following primary antibody incubation, tissue sections were washed 4× in PBS using baskets in 12 well plates for 5-min each wash. Tissues were then incubated in 200uL of secondary antibody solution containing a 1:500 dilution of the appropriate secondary antibody (Jackson Immunoresearch Alexafluor-488 and 549 secondaries) for 1.5 h in the dark at room temperature. If needed, after 1.5 h 300uL of a 1μg/mL DAPI solution was added to each tissue and incubated in the dark for 5 min at room temperature. Tissues were then washed 4× in PBS for 5 min each and carefully placed on Millenia 2.0 slides (StatLab Medical Products Cat#318) using a histology brush (Fisher Scientific Cat#NC0344756). Excess water was allowed to evaporate for 15 min, then each slide was mounted with 100uL of Prolong Gold Antifade Reagent (Thermo Fisher Cat#P36930) and a 1 mm cover slip.

All imaging was done using a Zeiss LSM 700 confocal microscope at either 40× or 63× magnification with a 1 AU pinhole. Following imaging, images were processed using either Imaris BitPlane software or the Fiji distribution of ImageJ. An Imaris BitPlane surfaces algorithm (Bitplane AG, Zurich, Switzerland) was used to quantify the tangle size vs. TIA1 intensity recorded in Fig. 1. The Coloc2 plugin of Fiji was used to determine colocalization parameters recorded in Fig. 4 following background subtraction with a rolling ball radius of 50. Intensity plots for Figs. 4 and 5 were also measured and generated using ImageJ-Fiji; regions of interest were set across a line using the line tool, and then measured using analyze- > plot profile.

### Tau immunoprecipitation for mass spectrometry analysis

2.5 mo rTg4510 and uninduced transgenic mouse brains (*n* = 4/group) were extracted, slowly frozen by submersion in methanol on dry ice, and homogenized in RIPA lysis buffer (50 mM Tris-HCl, 150 mM NaCl, 1% Triton X-100, 0.1% SDS, 0.5% sodium deoxycholate, pH 6.8) supplemented with protease (Roche Cat#04693159001) and phosphatase inhibitor (Roche Cat#04906837001) cocktails. Tau was immunoprecipitated from 1 mg cortex lysate using 10 μg of the mouse monoclonal Tau13 antibody immobilized on Pierce Direct IP columns according to manufacturer’s instructions (Thermo Scientific, Cat#26148). The Tau13 IP eluates were then separated on a Novex 4–12% Bis-Tris polyacrylamide gel (Life Technologies, Cat#NP0323) and stained with Simply Blue Coomassie G-250 SafeStain (Life Technologies, Cat#LC6060). Whole gel lanes were then excised and shipped to the UMass Mass Spectrometry and Proteomics facility for analysis by LC-MS/MS.

### Proteomics, in gel digestion

Gel slices were cut into 1 × 1 mm pieces and placed in 1.5 ml eppendorf tubes with 1 ml of water for 30 min. The water was removed and 50ul of 250 mM ammonium bicarbonate was added. For reduction 20 μl of a 45 mM solution of 1, 4 dithiothreitol (DTT) was added and the samples were incubated at 50 C for 30 min. The samples were cooled to room temperature and then for alkylation 20 μl of a 100 mM iodoacetamide solution was added and allowed to react for 30 min. The gel slices were washed 2 X with 1 ml water aliquots. The water was removed and 1 ml of 50:50 (50 mM Ammonium Bicarbonate: Acetonitrile) was placed in each tube and samples were incubated at room temperature for 1 h. The solution was then removed and 200 ul of acetonitrile was added to each tube at which point the gels slices turned opaque white. The acetonitrile was removed and gel slices were further dried in a Speed Vac. Gel slices were rehydrated in 75 μl of 2 ng/μl trypsin (Sigma) in 0.01% ProteaseMAX Surfactant (Promega): 50 mM Ammonium Bicarbonate. Additional bicarbonate buffer was added to ensure complete submersion of the gel slices. Samples were incubated at 37C for 21 h. The supernatant of each sample was then removed and placed in a separate 1.5 ml eppendorf tube. Gel slices were further dehydrated with 100 ul of 80:20 (Acetonitrile: 1% formic acid). The extract was combined with the supernatants of each sample. The samples were then dried down in a Speed Vac. Samples were dissolved in 25 μl of 5% Acetonitrile in 0.1% trifluroacetic acid prior to injection on LC/MS/MS.

### LC/MS/MS on Q Exactive

A 3.0 μl aliquot was directly injected onto a custom packed 2 cm × 100 μm C18 Magic 5 μm particle trap column. Labeled peptides were then eluted and sprayed from a custom packed emitter (75 μm × 25 cm C18 Magic 3 μm particle) with a linear gradient from 95% solvent A (0.1% formic acid in water) to 35% solvent B (0.1% formic acid in Acetonitrile) in 90 min at a flow rate of 300 nanoliters per minute on a Waters Nano Acquity UPLC system. Data dependent acquisitions were performed on a Q Exactive mass spectrometer (Thermo Scientific) according to an experiment where full MS scans from 300 to 1750 m/z were acquired at a resolution of 70,000 followed by 10 MS/MS scans acquired under HCD fragmentation at a resolution of 17,500 with an isolation width of 1.6 Da. Raw data files were processed with Proteome Discoverer (version 1.4) prior to searching with Mascot Server (version 2.5) against the Uniprot database. Search parameters utilized were fully tryptic with 2 missed cleavages, parent mass tolerances of 10 ppm and fragment mass tolerances of 0.05 Da. A fixed modification of carbamidomethyl cysteine and variable modifications of acetyl (protein N-term), pyro glutamic for N-term glutamine, oxidation of methionine were considered. Search results were loaded into the Scaffold Viewer (Proteome Software, Inc.).

### Proteomic analysis

Quantitative proteomic analysis was performed using the total ion current (TIC) for proteins identified by LC-MS/MS normalized to the TIC level of TIA1 detected in each sample. Proteins identified in the TIA1−/− samples were considered to be nonspecific binding proteins to the IP antibody and excluded from all subsequent analyses. Gene lists of detected proteins were then uploaded into the Database for Annotation, Visualization and Integrated Discovery (DAVID) resource available via the NIH website.

The proteins identified in the tau binding proteome in WT C57BL/6 J cortex resulted in 6 identified clusters with enrichment FDR < 0.05, and each of the proteins was associated to the cluster(s) based on its membership in the clustered gene sets. A network was created by adding a connection between protein pairs sharing annotation clusters. Edge weights were determined as the number of shared annotation clusters between protein pairs with thicker edges representing stronger functional associations between proteins (the smallest number of clusters shared between any two proteins was 1, and the largest was 8). The resulting network was visualized using the software Gephi 0.8.2 and arranged using the Force Atlas 2 layout algorithm. The network was generated using the python programming language (Python Software Foundation), and the networkx, numpy, and pandas python packages.

### CoIP validation of mass spectrometry

Brains from 6 month old PS19 mice expressing P301S tau were extracted and freshly homogenized using 400uL IP lysis buffer (0.025 M Tris; 0.15 M NaCl; 0.001 M EDTA; 1% NP-40, 5% glycerol; pH 7.4) supplemented with protease inhibitor cocktail (Roche Cat#04693159001), phosphatase inhibitor cocktail (Roche Cat#04906837001), and 20 units RNase inhibitor (ThermoFisher Scientific #AM2964). 1 mg of each lysate was pre-cleared with 80uL Protein G Dynabeads, and 50uL of Protein G Dynabeads (Invitrogen #10004D) per lysate were prepared with either 10μg Tau13 according to manufacturer instructions. Lysates and beads were equivalently pre-cleared and prepared with 10μg normal mouse IgG. Pre-cleared lysates were immunoprecipitated with the antibody conjugated beads using gentle rotation overnight at 4 °C; protein was eluted by boiling in Bolt SDS-PAGE sample buffer for 10 min and analyzed using SDS-PAGE. Antibodies used in the western blot analysis are recorded in supplemental Table 2.

### Sarkosyl fractionation and immunoblotting

Extracted brain tissue from (*n* = 3) rTg4510 and (*n* = 3) uninduced Tg4510 control mice was weighed and placed in a Beckman centrifuge tube, polycarbonate thick wall (Cat#362305). Tissue was homogenized in 4× weight/volume of homogenization buffer (50 mM Tris; 275 mM NaCl; 5 mM KCl; 1 mM PMSF; pH = 8.0 with protease inhibitors, phosphatase inhibitors and PMSF added immediately before use) and ultracentrifuged at 28 k rpm (29,800 g) in a TLA-55 rotor for 20 min at 4 °C using a Beckman Optima-TLX 120,000 ultracentrifuge. The supernatant was removed and stored at − 80 °C as the TBS soluble supernatant (supernatant S1); excess supernatant was then vacuumed off the pellet, and the pellet was suspended in sucrose buffer (10 mM Tris, pH = 7.4; 0.8 M NaCl; 10% sucrose; 1 mM EGTA; 1 mM PMSF). The suspension was ultracentrifuged at 22 k rpm (26,300 g) for 20 min at 4 °C. 450uL of the supernatant was transferred to a new tube with the pellet stored at − 80 °C (pellet P2). This supernatant was incubated with 1% Sarkosyl for 5 min with gentle rotation at room temperature then 1 h at 37 °C. After Sarkosyl incubation the solutions were ultracentrifuged at 55 k rpm (150,000 g) for 1 h at 4 °C. The supernatant was stored at − 80 °C (supernatant S3) and the pellet was resuspended in 60uL TE buffer (10 mM Tris, pH = 8.0, 1 mM EDTA) and also kept at − 80 °C as the P3 pellet.

The Sarkosyl soluble S3 supernatant and insoluble P3 pellet were analyzed for RBP content using SDS-PAGE and immunoblotting. As the protein concentration of all tissues were normalized at the start of fractionation, 20uL of the P3 pellet and 5uL of the S3 supernatant were run on a Bolt 4–12% Bis-Tris Plus polyacrylamide gel (Life Technologies Cat#NW04127BOX) at 120 V for 1 h and 15 min. Proteins were transferred onto nitrocellulose at 12 V for 1 h, and membranes were blocked in Tris-buffered saline (24 g Tris base; 88 g NaCl; 1 L water; pH = 7.6) with 0.1% Tween (TBS-T); with 5% non-fat milk for 2 h. After blocking membranes were incubated with 10 mL of the appropriate primary antibody in TBS-T overnight at 4 °C; primary antibodies and dilutions are recorded in supplementary Table 2. Membranes were washed 4× for 5 min each was in TBS-T, then incubated with the appropriate HRP-conjugated secondary antibody at a 1:15,000 dilution in TBS-T (Jackson Immunoresearch) for 1.5 h. Membranes were again washed 4× for 5 min each in TBS-T then developed using Pierce SuperSignal West Pico Chemiluminescent Substrate (Thermo Fisher Scientific Cat#34080). Blots were quantified using ImageJ peak analysis.

## Additional files

Additional file 1: **Figure S1.** TIA1 antibodies demonstrate significant variability. Four commercial TIA1 antibodies were screened for performance in fluorescent immunohistochemical assays: Abcam 40,693 (A), Santa Cruz 1751 (B), Abcam 140,595 (C), and Cell Signaling 1398S (D). Of these, only Abcam 40,693 demonstrated affinity for both cytoplasmic and nuclear TIA1 with minimal background reactivity, but also shows performance variability between lots (E). **Figure S2**. Co-immunoprecipitation (co-IP) and immunoblotting (IB) of tau and RBPs from the brains of P301S tau mice (*n* = 2). HNRNPA0 (35kD), eIF4a2 (47kD), HNRNPK (55kd), and RPL11 (23kD) co-IPd with Tau13 (left) but not normal mouse IgG (right). **Figure S3.** Immunhistochemical analysis of rTg4510 tissue (*n* = 3) revealed a significant co-localization in the cortex between the PCBP2 (*r* = 0.724), RPL11 (*r* = 0.728), and eIF3h (*r* = 0.315) (red) with pathological phospho-tau stained with CP13 antibody (green). **Figure S4.** Duration of fixation affects sensitivity of RBP detection. Samples were fixed for 24 h (top row) or 48 h (bottom row) with 4%, and imaged for NeuN or TIA1; DAPI identifies nuclei. **Figure S5.** Photobleaching of tissue removes autofluorescence from lipofuscin and the extracellular matrix. Human AD tissue was treated with white light from an LED bulb for 72 h and then imaged. Untreated tissue shows significant autofluoresence in the red and green channels (top), which was removed with photobleaching (bottom). **Figure S6.** Consolidated but not diffuse phospho-tau is present in late stage tissue. Tangle morphology and intensity were compared in 6-month rTg4510 mouse tissue (left) and human AD tissue (right). In the human tissue, CP13 positive tau presents entirely as consolidated NFTs, which extend into the processes. The mouse tissue showed a continuum of pathological tau including diffuse cytoplasmic phospho-tau (white arrows), CP13 positive puncta, and intense, consolidated NFTs. (PDF 956 kb)

Additional file 2: **Table S1.** Mass spectometry data. This table provides quantification of the proteins identified by mass spectrometry, and shows # peptides identified, fold changes and *P*-values for each protein identified. (XLSX 65 kb)

Additional file 3: **Table S2.** List of antibodies used in the study. This table provides source information for each antibody, as well as the diltuion at which each antibody was used in the experiments. (XLSX 9 kb)
