# Supplementary material for: RNA binding proteins co-localize with small tau inclusions in tauopathy
Source: Acta Neuropathol Commun. 2018 Aug 1;6:71. doi: 10.1186/s40478-018-0574-5 (PMC6069705; doi:10.1186/s40478-018-0574-5)

Supplemental Figure 1.

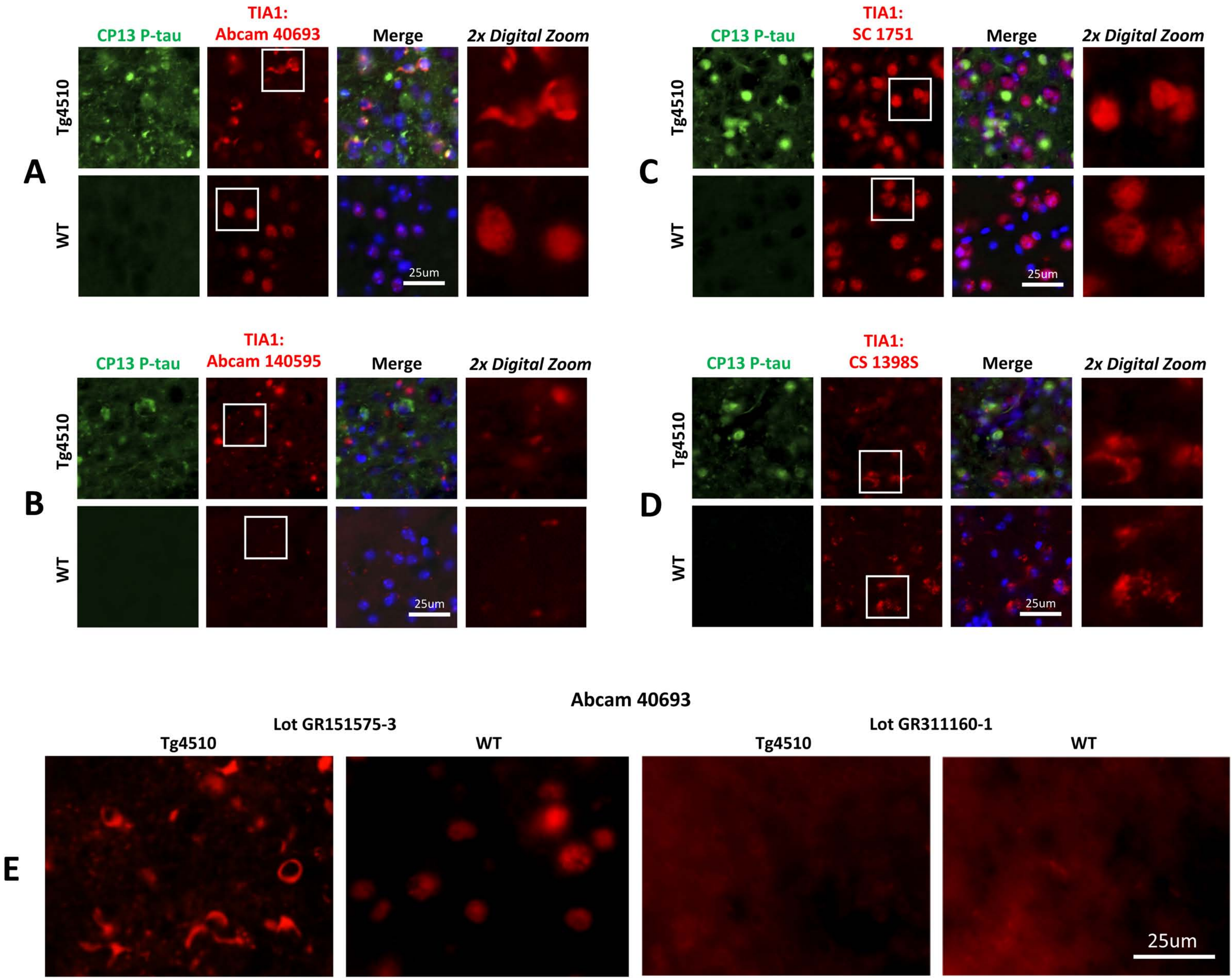

Supplemental Figure 2.

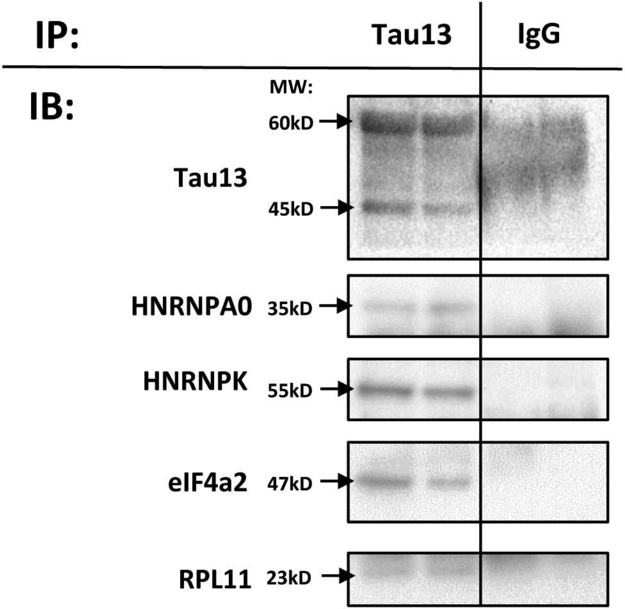

Supplemental Figure 3.

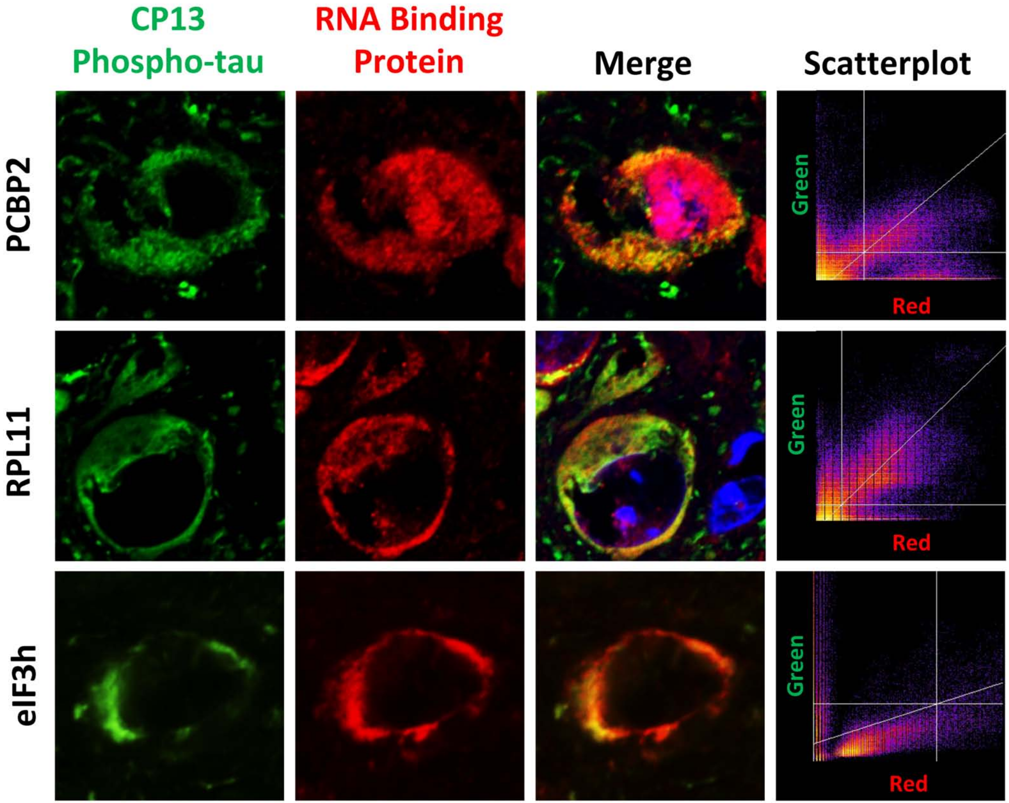

Supplemental Figure 4.

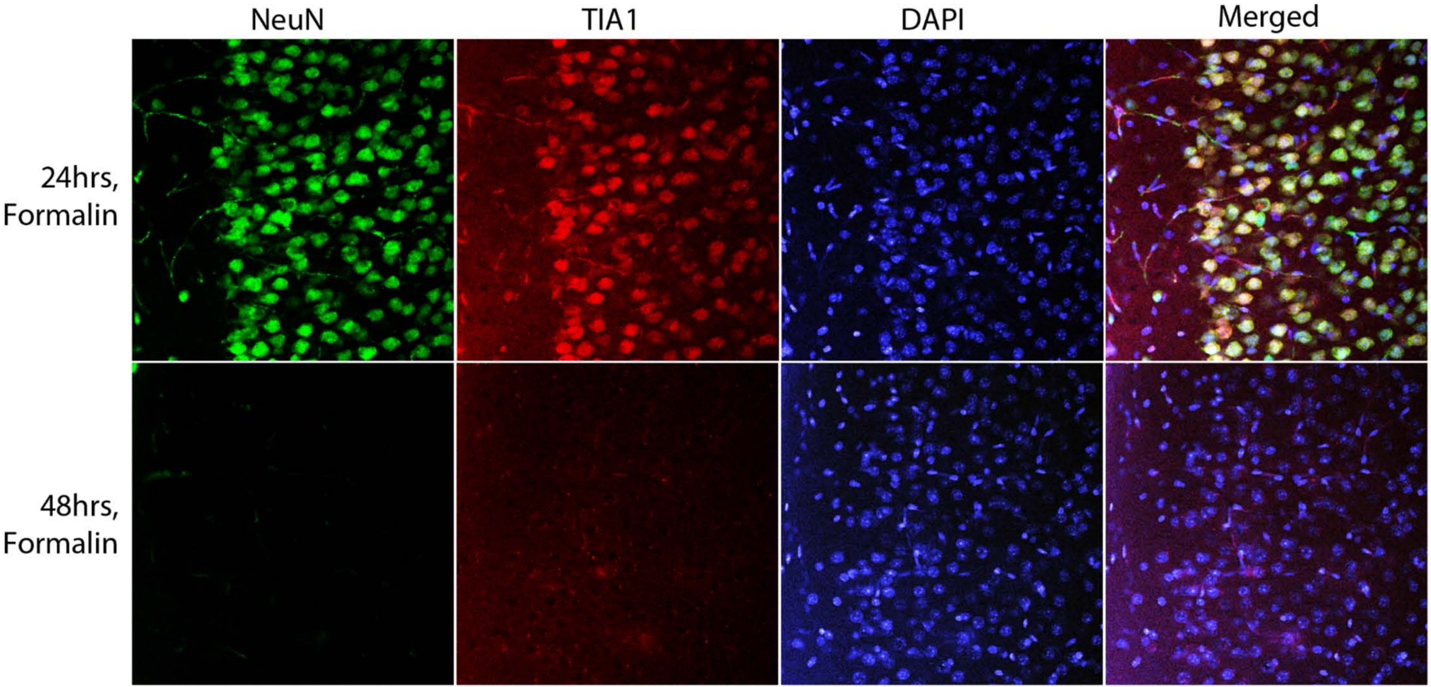

Supplemental Figure 5.

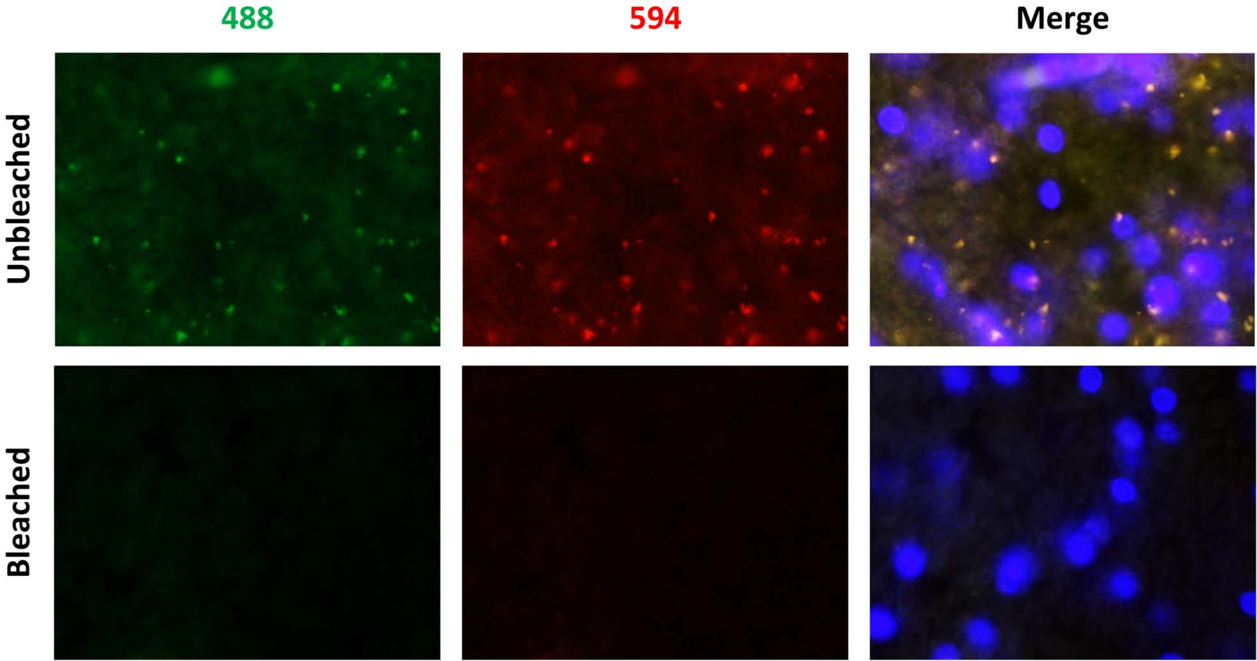

Supplemental Figure 6.

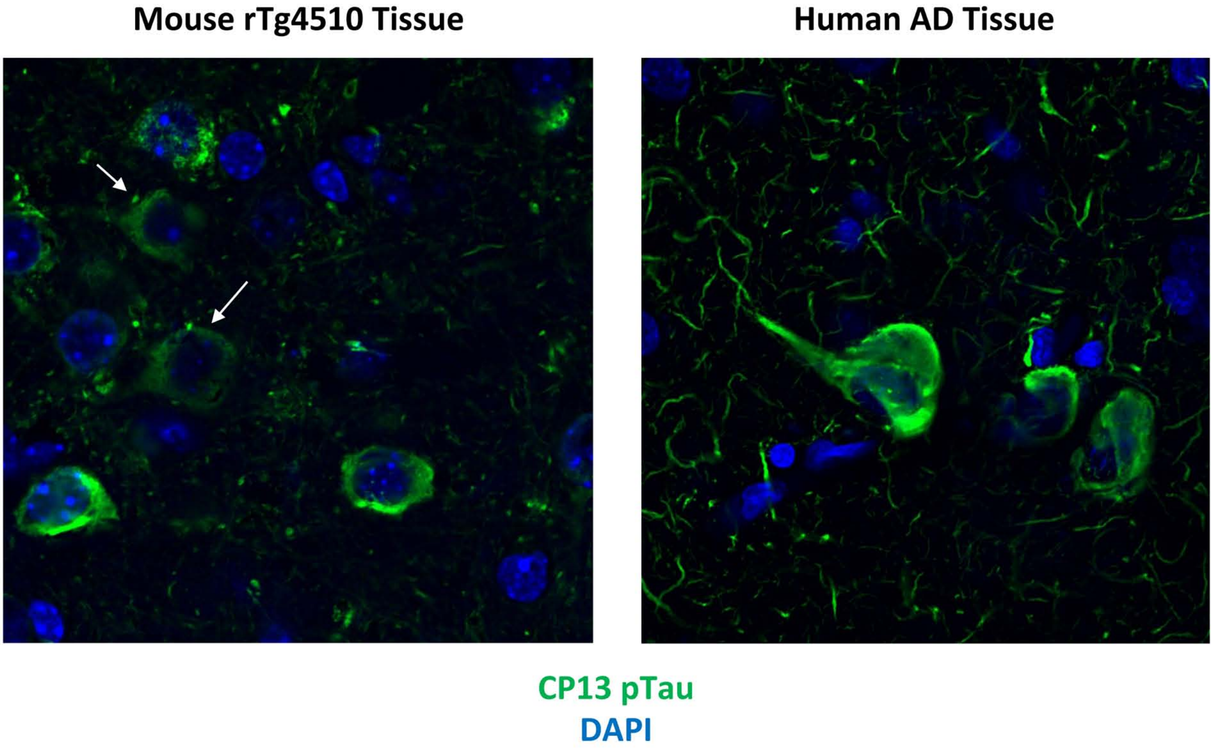

Supplement: Supplementary file 1 — Figure S1. TIA1 antibodies demonstrate significant variability. Four commercial TIA1 antibodies were screened for performance in fluorescent immunohistochemical assays: Abcam 40,693 (A), Santa Cruz 1751 (B), Abcam 140,595 (C), and Cell Signaling 1398S (D). Of these, only Abcam 40,693 demonstrated affinity for both cytoplasmic and nuclear TIA1 with minimal background reactivity, but also shows performance variability between lots (E). Figure S2. Co-immunoprecipitation (co-IP) and immunoblotting (IB) of tau and RBPs from the brains of P301S tau mice (n = 2). HNRNPA0 (35kD), eIF4a2 (47kD), HNRNPK (55kd), and RPL11 (23kD) co-IPd with Tau13 (left) but not normal mouse IgG (right). Figure S3. Immunhistochemical analysis of rTg4510 tissue (n = 3) revealed a significant co-localization in the cortex between the PCBP2 (r = 0.724), RPL11 (r = 0.728), and eIF3h (r = 0.315) (red) with pathological phospho-tau stained with CP13 antibody (green). Figure S4. Duration of fixation affects sensitivity of RBP detection. Samples were fixed for 24 h (top row) or 48 h (bottom row) with 4%, and imaged for NeuN or TIA1; DAPI identifies nuclei. Figure S5. Photobleaching of tissue removes autofluorescence from lipofuscin and the extracellular matrix. Human AD tissue was treated with white light from an LED bulb for 72 h and then imaged. Untreated tissue shows significant autofluoresence in the red and green channels (top), which was removed with photobleaching (bottom). Figure S6. Consolidated but not diffuse phospho-tau is present in late stage tissue. Tangle morphology and intensity were compared in 6-month rTg4510 mouse tissue (left) and human AD tissue (right). In the human tissue, CP13 positive tau presents entirely as consolidated NFTs, which extend into the processes. The mouse tissue showed a continuum of pathological tau including diffuse cytoplasmic phospho-tau (white arrows), CP13 positive puncta, and intense, consolidated NFTs. (PDF 956 kb) [file 40478_2018_574_MOESM1_ESM.pdf]
